# Impaired insulin receptor function alters psychiatric behaviors without affecting circadian rhythms in a mouse model of Alzheimer's disease

**DOI:** 10.1111/ggi.70093

**Published:** 2025-05-29

**Authors:** Naotaka Izuo, Koutaro Yokote, Takahiko Shimizu

**Affiliations:** ^1^ Department of Endocrinology, Hematology and Gerontology, Graduate School of Medicine Chiba University Chiba Japan; ^2^ Research Center for Advanced Science and Technology The University of Tokyo Tokyo Japan; ^3^ Aging Stress Response Research Project Team National Center for Geriatrics and Gerontology Obu Japan; ^4^ Department of Food and Reproductive Function Advanced Research Juntendo University Graduate School of Medicine Tokyo Japan

**Keywords:** Alzheimer's disease, anxiety, BPSD, depression, insulin resistance

## Abstract

**Aim:**

Behavioral and psychological symptoms of dementia (BPSD) are a cause of reduced quality of life in patients with Alzheimer's disease (AD) and their caregivers. In this study, we examined the influence of insulin resistance, a risk factor for AD, on BPSD in AD pathology and related adrenergic and dopaminergic gene expressions in a mouse model.

**Methods:**

Constitutive knock‐in mice with the P1195L mutation in the insulin receptor (IR‐KI mice) and those with a mutated amyloid precursor protein (*App* ^*NL‐G‐F*^ mice: APP‐KI mice) were crossbred to obtain the resultant mice (APP/IR‐dKI mice) as AD models with insulin resistance for behavioral analysis.

**Results:**

APP/IR‐dKI mice exhibited significantly longer immobilization in the forced swimming test and longer time spent on the light side in the light/dark box test than did APP‐KI mice. No differences in circadian rhythms and physical activity in the dark and light phases were observed between APP‐KI and APP/IR‐dKI mice. Regarding gene expression of *Adr1a1*, *Adr1a2*, *Adr1ad*, *Drd1*, and *Drd2* in the cortical region, there were no differences between APP‐KI and APP/IR‐dKI mice. These results suggest that APP/IR‐dKI mice exhibit enhanced depression and suppressed anxiety compared with APP‐KI mice, without mediation by the alteration of adrenergic or dopaminergic receptors.

**Conclusions:**

Insulin resistance could enhance depression and suppress anxiety in relation to BPSD in AD. **Geriatr Gerontol Int 2025; 25: 967–971**.

## Graphical abstract

A mouse model of Alzheimer's disease (AD) with genetically induced insulin resistance without hyperglycemia exhibits depressive and anxiolytic behaviors. Insulin resistance could enhance depression and suppress anxiety in relation to the behavioral and psychological symptoms of dementia in AD.

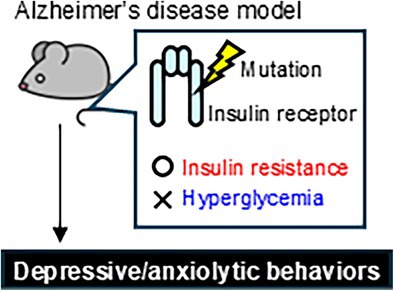

## Introduction

Alzheimer's disease (AD) is a neurodegenerative disease characterized by cognitive dysfunction such as memory impairment and reasoning deficits. In addition, behavioral and psychological symptoms of dementia (BPSD) worsen the quality of life (QOL) of patients and caregivers.^1^ BPSD includes anxiety, depression, apathy, agitation, and aggression.^1^, ^2^, ^3^ Approximately 75% patients with dementia exhibit at least one BPSD after the onset of cognitive symptoms according to a population‐based study.^4^ As BPSD symptoms become more severe along with AD progression, the benefits of medication become limited. An understanding of BPSD contributes to social welfare and individual well‐being. However, the factors contributing to the diversity of BPSD remain unclear. Considering that AD pathology is modified by various risk factors, such as metabolic syndrome, psychological stress, and dietary patterns,^5^, ^6^, ^7^, ^8^, ^9^ the diversity of BPSD could be derived from a variety of risk factors. Recently, our group demonstrated that insulin resistance, one of the risk factors for AD,^10^, ^11^, ^12^ induces an earlier onset of cognitive dysfunction in a mouse model of AD.^13^ In that study, constitutive knock‐in mice with the P1195L mutation in the insulin receptor (IR‐KI mice)^14^, ^15^ and those with mutated amyloid precursor protein (*App* ^*NL‐G‐F*^ mice: APP‐KI mice)^16^, ^17^ were crossbred, and the resultant mice (APP/IR‐dKI mice) were analyzed. APP/IR‐dKI mice exhibited insulin resistance and an earlier onset of cognitive dysfunction than APP‐KI mice.^13^ This experimental framework using mouse models can be applied to understanding the relationship between insulin resistance and BPSD. Therefore, the present study analyzed the psychiatric behaviors and related adrenergic and dopaminergic gene expression of APP/IR‐dKI mice.

## Materials and methods

### Animals

Experimental animals were maintained at 24 ± 1°C and 55 ± 10% relative humidity under a 12‐h light/dark cycle, with *ad libitum* access to standard rodent chow and water. In this study, APP‐KI, IR‐KI, and APP/IR‐dKI mice represented APP (homozygote)‐KI, IR (heterozygote)‐KI, and APP (homozygote)/IR (heterozygote)‐dKI mice, respectively. APP‐KI and APP/IR‐dKI mice were produced by breeding male APP (homozygote)/IR (heterozygote)‐dKI mice with female APP (homozygote)‐KI mice because female APP (homozygote)/IR (heterozygote)‐dKI mice frequently kill their newborn pups, possibly owing to let‐down reflex dysfunction. Male mice were used in all experiments because female IR‐KI mice exhibit a weak insulin‐resistant phenotype owing to estrogen.^14^, ^15^ Behavioral analyses were performed at the age of 4 months.

### Behavioral tests

#### Forced swimming test

The forced swimming test was performed as described in a previous study.^18^ In brief, the mice were placed in an open‐topped transparent polycarbonate box (27 cm × 44 cm × 20 cm (height)) containing water at 22°C at a depth of 18 cm. The mice were forced to swim for 6 min. The duration of immobility was measured over the final 4 min.

#### Light/dark box test

The light/dark box test was performed as described in a previous study.^19^ In brief, to evaluate the anxiety response to the light environment, the mice were allowed to explore the apparatus, which consisted of an open‐topped “light” compartment and a close‐topped “dark” compartment, for 5 min. The total time spent in each compartment was recorded.

#### Spontaneous activity test

Following a previous study,^20^ spontaneous activity was measured using an animal movement analysis system (ACTIMO system, Bio Research Center., Aichi, Japan), which consisted of a rectangular enclosure (30 × 20 cm) with a side wall equipped with photo sensors at 2‐cm intervals. Each pair of photo sensors scanned animal movement at 0.5‐s intervals. Mice were singly housed in a cage, and measurements were started after a 7‐day habituation period. Circadian rhythms and spontaneous activity during the dark and light phases were calculated as an average of the measurements over 14 days.

#### Quantitative reverse transcription polymerase chain reaction

Mice were administered anesthesia through intraperitoneal injection of three mixed anesthetics (0.75 mg/kg domitol, Nippon Zenyaku Kogyo, Fukushima, Japan; 4 mg/kg midazolam, SANDOZ, Tokyo, Japan; and 5 mg/kg butorphanol, Meiji Seika Pharma, Tokyo, Japan) and then perfused with ice‐cold phosphate‐buffered saline (PBS) to remove their blood. The cortical regions were collected and placed in TRIzol RNA Isolation Reagent (Thermo Fisher Scientific, MA, USA) for RNA extraction. Tissues were homogenized in TRIzol reagent. The total RNA was extracted according to the manufacturer's instructions. cDNA was synthesized using a ReverTra Ace qRT‐PCR kit (TOYOBO, Osaka, Japan). Total cDNA (100 ng) was used as the template for qRT‐PCR analysis. cDNA was quantified using an ABI Prism 7500 sequence‐detection system with the primers and SYBR Green PCR Master Mix (Thermo Fisher Scientific), according to the manufacturer's instructions. The detector was programmed with the following PCR conditions: 40 cycles of denaturation at 95°C for 15 s each and 1 min of amplification at 56.5°C. *Actb* was used as the internal control. Relative differences in PCR results were calculated using the comparative cycle threshold method. Primers used in this study were as follows: *Adra1a* forward: 5′‐ttttcttggctaacagacaggc‐3′, reverse: 5′‐gtaacaatgcctcttggaaaaacac‐3′; *Adra1b* forward: 5′‐atacctgggtcgtggaacg‐3′, *reverse*: 5′‐ggagcttgaaagtgaagagtgg‐3′; *Adra1d* forward: 5′‐cgctgtggtgggaaccggcag‐3′, reverse: 5′‐acagctgcactcagtagcaggtca‐3′; *Drd1* forward: 5′‐ccttcgatgtatttgtgtgg‐3′, reverse: 5′‐agcatgagggatcaggtaaa‐3′; *Drd2* forward: 5′‐aactgtacccaccctgagga‐3′, reverse: 5′‐gttgctatgtagaccgtg‐3′, *Actb* forward: 5′‐tgccctgaggctcttttcca‐3′, and reverse, 5′‐ttggcatagaggtctttacggat‐3′.

#### Statistics

All data are presented as the mean ± standard error of the mean (SEM). Statistical differences between the two groups were determined using Student's *t*‐test. All statistical analyses were performed using Prism, version 9 (GraphPad Software, CA, USA).

## Results

**Figure 1:**
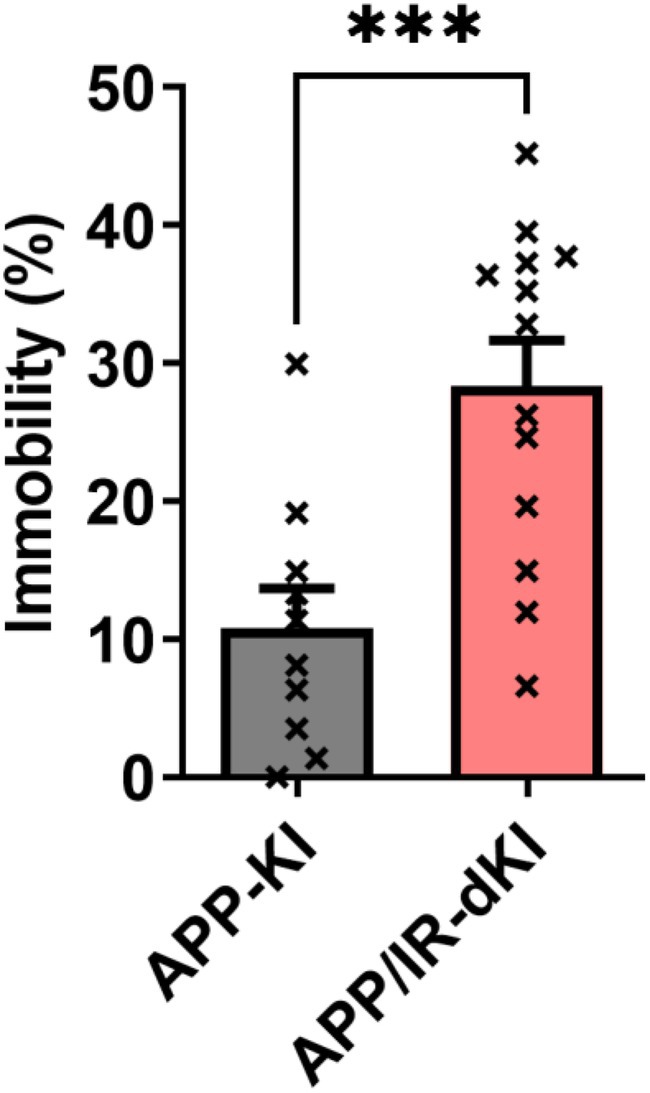
APP/IR‐dKI mice exhibit enhanced depression behavior in the forced swimming test. The immobility time of APP/IR‐dKI mice was significantly longer than that of APP‐KI mice in the forced swimming test (APP‐KI mice, *n* = 10; APP/IR‐dKI mice, *n* = 13; ***P = 0.0009). Values represent the mean ± SEM.

**Figure 2:**
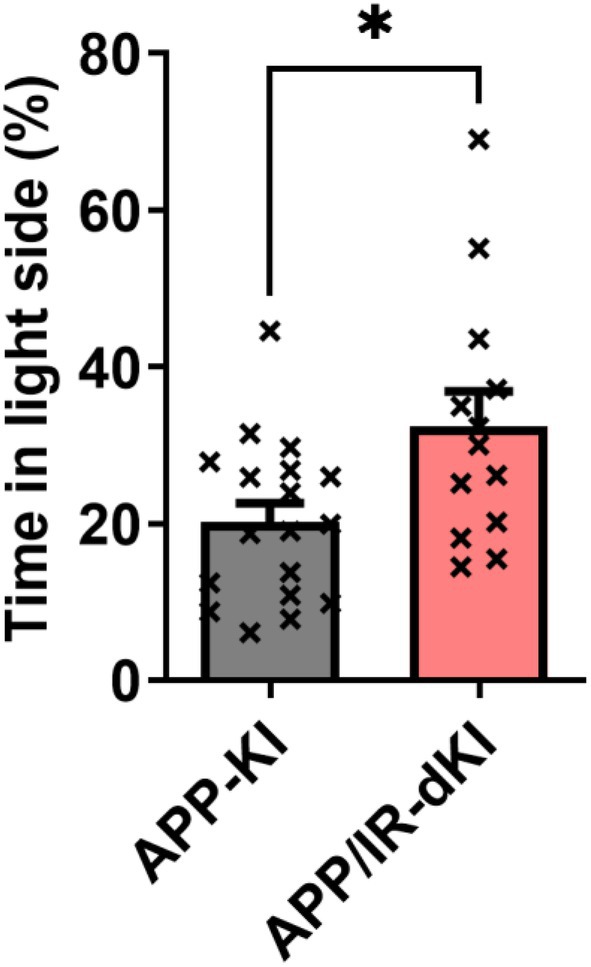
APP/IR‐dKI mice exhibit enhanced depression behavior in the light/dark box test. The time spent by APP/IR‐dKI mice on the light side was significantly longer than that spent by APP‐KI mice in the light/dark box test (APP‐KI mice, *n* = 18; APP/IR‐dKI mice, *n* = 13; *P = 0.0144). Values represent the mean ± SEM.

**Figure 3:**
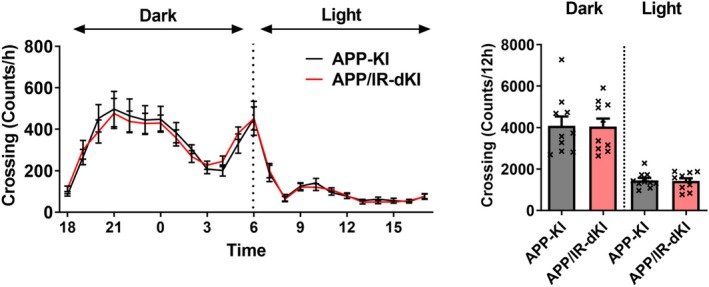
APP/IR‐dKI mice did not exhibit an alteration of circadian rhythm or physical spontaneous activity compared with APP‐KI mice. (a, b) In the animal movement analyzing system, no difference was observed in the dark phase or light phase of physical spontaneous activity between APP/IR‐dKI and APP‐KI mice (APP‐KI mice, *n* = 10; APP/IR‐dKI mice, *n* = 10; *P* = 0.9574 in dark phase; *P* = 0.8667 in the light phase). Values represent the mean ± SEM.

**Figure 4:**
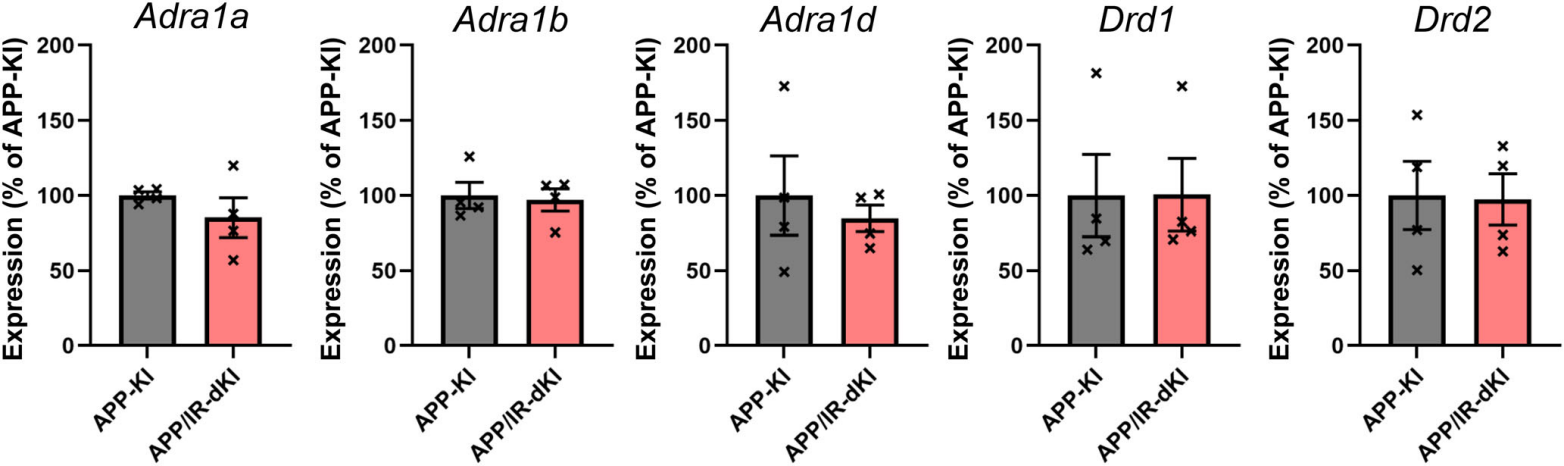
APP/IR‐dKI mice did not exhibit an alteration of gene expression of adrenergic or dopaminergic receptors compared with APP‐KI mice. In the qRT‐PCR, no difference was found in gene expression levels of *Adra1a* (*P* = 0.313), *Adra1b* (*P* = 0.805), *Adra1d* (*P* = 0.605), *Drd1* (*P* = 0.988), and *Drd2* (*P* = 0.929) between APP‐KI (*n* = 4) and APP/IR‐dKI (*n* = 4) mice. Expression levels of each gene were normalized by those of *Actb*. Values represent the mean ± SEM.

The depression behavior of APP/IR‐dKI mice was evaluated at the age of 4 months. APP/IR‐dKI mice exhibited a significantly longer immobilization time than APP‐KI mice in the forced swimming test (***P = 0.0009, APP‐KI [*n* = 10] vs. APP/IR‐dKI [*n* = 13]; Fig. 1). We also confirmed that IR‐KI mice did not exhibit a longer immobilization time (Fig. S1). Anxiety of APP/IR‐dKI mice was examined in the light/dark box test. APP/IR‐dKI mice spent a significantly longer time in the light side than did APP‐KI mice (*P = 0.0144, APP‐KI [*n* = 18] vs. APP/IR‐dKI [*n* = 13]; Fig. 2). The daily activities of APP‐KI and APP/IR‐dKI mice were compared. No abnormalities were observed in the circadian rhythms in APP‐KI or APP/IR‐dKI mice. There were no differences in the activity in the dark or light phase between APP‐KI and APP/IR‐dKI mice (*P* = 0.9574 in the dark phase and *P* = 0.8667 in the light phase; APP‐KI [*n* = 10] vs. APP/IR‐dKI [*n* = 10]; Fig. 3). These results suggest that APP/IR‐dKI mice exhibit enhanced depression and suppressed anxiety compared with APP‐KI mice. To understand the underlying mechanism of behavioral alteration, genetic expressions of adrenergic and dopaminergic receptors in the cortical region were examined (Fig. 4). There were no significant differences in the expression of *Adra1a* (*P* = 0.313), *Adra1b* (*P* = 0.805), *Adra1d* (*P* = 0.605), *Drd1* (*P* = 0.988), and *Drd2* (*P* = 0.929) between APP‐KI and APP/IR‐dKI mice.

## Discussion

Our group has clarified the effect of insulin resistance on AD pathology using AD model mice harboring the P1195L mutation in the insulin receptor, which diminishes the receptor tyrosine kinase activity to induce insulin resistance.^14^, ^15^ Just recently, it was reported that APP/IR‐dKI mice, which show insulin resistance without persistent hyperglycemia, exhibit earlier onset of cognitive impairment and no difference in Aβ accumulation compared with APP‐KI mice.^13^ In the present study, APP/IR‐dKI mice also exhibited enhanced depression and anxiety compared with APP‐KI mice. These findings suggest that insulin resistance, independently of hyperglycemia, affects the cognitive and psychological symptoms of AD. There is limited literature on the relationship between the BPSD of AD and its risk factors. Sakurai *et al*. sought links between subtypes of older diabetic patients with AD and their clinical features and found a higher prevalence of physical and psychological complications in AD patients with higher HbA1c.^21^ In that study, no correlation was found between the status of the Geriatric Depression Scale‐15 and HbA1c.^21^ In contrast, Shi *et al*. reported a higher prevalence of T2DM in AD patients with depression or anxiety than in those without corresponding psychological symptoms.^22^ T2DM includes insulin resistance and hyperglycemia. As hyperglycemia increases Aβ production, it modifies AD pathology independently of insulin resistance.^23^, ^24^ To understand the relationship between BPSD and T2DM, a clinical investigation of the link between each index of T2DM and BPSD status is necessary.

Higher depression and lower anxiety behaviors were observed in the mouse model of AD with systemic insulin resistance. In contrust, Kleinridders *et al*. reported that both depression and anxiety behaviors were enhanced in the brain‐specific knockout of the insulin receptor (NIRKO mice).^19^ From the comparison of these findings, depression and anxiety behaviors related to insulin resistance could be differently regulated. The higher depression behavior in APP/IR‐dKI mice may be induced by insulin resistance, especially in the brain, and the lower anxiety behavior may be elicited by peripheral insulin resistance. For further understanding of the underlying mechanism, gene expression levels of adrenergic and dopaminergic receptors, which are reported to be involved in depression and anxiety,^25^, ^26^, ^27^ were examined. There were no differences in expression levels of *Adr1a1*, *Adr1a2*, *Adr1ad*, *Drd1*, and *Drd2* between APP‐KI and APP/IR‐dKI mice, suggesting that the behavioral changes of APP/IR‐dKI mice are not mediated by the alteration of these receptors, but possibly by the abnormality of synaptic regulation or neurotransmitter metabolism. Considering that NIRKO mice exhibit deregulation of the insulin‐dependent modulation of dopamine metabolism^19^ and that therapeutics targeting dopamine and serotonin neurotransmission are generally used for BPSD symptoms of AD patients, further studies focusing on neurotransmitters will lead to an understanding of the underlying mechanism of abnormal behavior in APP/IR‐dKI mice. As mentioned in the Materials and methods section, female APP/IR‐dKI mice exhibited let‐down reflex dysfunction. A similar phenotype has been reported in oxytocin‐deficient mice,^28^ which also exhibit milk‐ejection abnormalities. In addition, evidence that the administration of oxytocin exerts an antidepressant effect^29^ and, in certain contexts, enhances anxiety^30^, ^31^ is consistent with our behavioral results. Therefore, oxytocin should be explored as a mechanistic factor for behavioral alterations in APP/IR‐dKI mice.

Through the analyses of APP/IR‐dKI mice, we have clarified the relationship between insulin resistance and BPSD. Elucidating the clinical features and mechanisms of AD patients with insulin resistance would contribute to the effective medication of BPSD to improve the QOL of patients and their caregivers.

## Funding information

This study was supported by Japan Society for the Promotion of Science (JSPS) KAKENHI (Grants 15K19279, 20K16006, 23K07007 to NI), a Grant‐in‐Aid for JSPS Fellows (Grant 16J05570 to NI), and by Research Funding for Longevity Sciences (19‐50 to TS) from the National Center for Geriatrics and Gerontology.

## Disclosure statement

The authors declare no conflict of interest.

## Author contributions

NI and TS designed the study. NI wrote the manuscript. NI performed the experiments and analyzed the data. NI, KY, and TS discussed the hypothesis and interpreted the data. TS and KY provided critical suggestions. NI, KY, and TS edited the manuscript. NI and TS coordinated and directed the project.

## Ethics statement

All experimental procedures were performed in accordance with the specified guidelines for the care and use of laboratory animals and were approved by the Animal Care and Use Committee of Chiba University (approval number: 1‐282), the National Center for Geriatrics and Gerontology (approval number: 2‐24), the Committee for Recombinant DNA Experiments at Chiba University (approval number: 28‐79), and the National Center for Geriatrics and Gerontology (approval number: 2‐28).

## Supporting information


**Data S1.** Supporting Information.

## Data Availability

The data that support the findings of this study are available from the corresponding author upon reasonable request.
