# Supplementary material for: Impaired insulin receptor function alters psychiatric behaviors without affecting circadian rhythms in a mouse model of Alzheimer's disease
Source: Geriatr Gerontol Int. 2025 May 29;25(7):967–71. doi: 10.1111/ggi.70093 (PMC12238808; doi:10.1111/ggi.70093)
Supplement: Supplementary file 1 — Data S1. Supporting Information. [file GGI-25-967-s001.pdf]

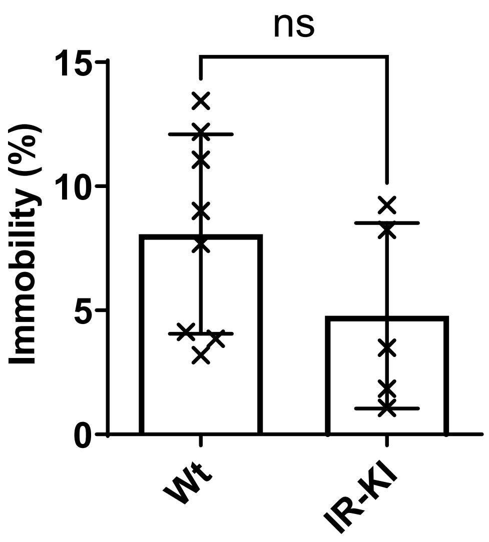

Figure 1. IR-KI mice did not exhibit depression behavior in the forced swimming test.

There was no significant difference in immobility time between Wt and IR-KI mice (Wt mice, n = 8; IR-KI mice, n = 5; p = 0.168). Values represent the mean ± S. E. M.
